# Generation of chickens expressing Cre recombinase

**DOI:** 10.1007/s11248-016-9952-6

**Published:** 2016-03-31

**Authors:** Philip A. Leighton, Darlene Pedersen, Kathryn Ching, Ellen J. Collarini, Shelley Izquierdo, Roy Jacob, Marie-Cecile van de Lavoir

**Affiliations:** 1 Crystal Bioscience, 5980 Horton Street, Suite 405, Emeryville, CA 94608 USA; 2 Bayer Animal Health, 12707 Shawnee Mission Pkwy, Shawnee, KS 66216 USA

**Keywords:** Transgenic chicken, Cre recombinase, Primordial germ cell

## Abstract

Cre recombinase has been extensively used for genome engineering in transgenic mice yet its use in other species has been more limited. Here we describe the generation of transgenic chickens expressing Cre recombinase. Green fluorescent protein (GFP)-positive chicken primordial germ cells were stably transfected with β-actin-Cre-recombinase using phiC31 integrase and transgenic chickens were generated. Cre recombinase activity was verified by mating Cre birds to birds carrying a floxed transgene. Floxed sequences were only excised in offspring from roosters that inherited the Cre recombinase but were excised in all offspring from hens carrying the Cre recombinase irrespective of the presence of the Cre transgene. The Cre recombinase transgenic birds were healthy and reproductively normal. The Cre and GFP genes in two of the lines were closely linked whereas the genes segregated independently in a third line. These founders allowed development of GFP-expressing and non-GFP-expressing Cre recombinase lines. These lines of birds create a myriad of opportunities to study developmentally-regulated and tissue-specific expression of transgenes in chickens.

## Introduction

Throughout the nineteenth and twentieth centuries, chick embryos were a major model for vertebrate development. However, the chicken’s preeminent position was gradually lost when ES cells became available in mice, allowing rapid manipulation of genes and evaluation of resulting phenotypes. In the last few years, however, tools to create transgenic chickens have been developed (Mcgrew et al. 2004; Van De Lavoir et al. 2006; Macdonald et al. 2012; Park and Han 2012; Tyack et al. 2013) and more transgenic lines will become available.

Our group has produced transgenic chickens using primordial germ cells (PGCs) as intermediates (Van De Lavoir et al. 2006; Schusser et al. 2013; Collarini et al. 2015). The transgenes are inserted into the PGC genome with a selection cassette that includes a gene conferring antibiotic resistance. It has been reported that strong and ubiquitous promoters can interfere with expression of endogenous and adjacent loci (Fiering et al. 1995; Olson et al. 1996), leading to the conclusion that it is preferable to remove selection cassettes from lines of transgenic animals.

Cre is a bacteriophage P1 recombinase that has been widely used in genome engineering, conditional knockouts, and the regulation of transgene expression in more than 450 lines of transgenic mice (http://jaxmice.jax.org/list/xprs_creRT1801.html). It catalyzes recombination between 34 bp loxP DNA elements consisting of two 13 bp inverted repeats separated by a directional 8 bp core. When mice expressing Cre recombinase are mated to mice carrying a gene that is flanked by loxP sites (i.e. floxed), the intervening DNA is excised. Cre recombinase can be expressed ubiquitously and the floxed alleles are excised in all tissues, including the germline (Nagy 2000). In other cases, Cre recombinase is used to confer tissue-specific and developmentally-regulated gene expression by placing the Cre gene under the control of a tissue-specific promoter. In general, loxP sites are the target of Cre recombinase although off-target effects have been recorded both in vitro (Thyagarajan et al. 2000; de Alboran et al. 2001; Loonstra et al. 2001) and in vivo (Schmidt et al. 2000; Higashi et al. 2009).

In this manuscript we describe the generation of lines of chickens that express Cre recombinase. Primordial germ cells were transfected with Cre recombinase under the control of the β-actin promoter, selected for neomycin resistance, expanded and injected into recipient embryos. The resulting G0 chimeras were grown to sexual maturity and bred to produce G1 offspring. The G1 birds carrying Cre recombinase were bred to transgenic chickens carrying a floxed transgene. Offspring from the Cre roosters showed recombination of the floxed transgene when Cre recombinase was inherited from the father. In contrast, all offspring from the Cre hens, irrespective of the inheritance of the Cre recombinase gene, showed a recombined transgene, indicating that sufficient Cre recombinase is present in the cytoplasm of the oocyte to recombine the transgenes that enter at fertilization.

## Results

### Production of stably transfected primordial germ cells

**Fig. 1:**
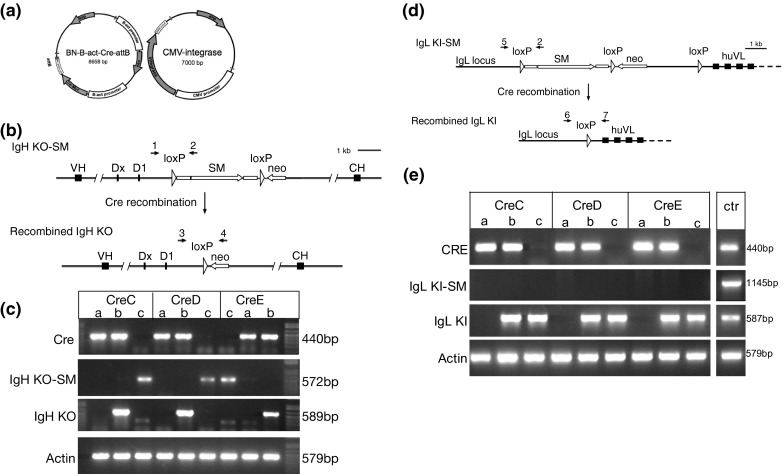
Generation of Cre recombinase transgenic chickens and removal of a selectable marker cassette. **a** PGCs were co-transfected with a β-actin Cre (β-actin-Neo, β-actin-Cre-recombinase, attB) and a CAG-integrase plasmid to generate stable Cre recombinase clones. **b** A schematic showing the heavy chain immunoglobulin knockout locus containing a selectable marker cassette (SM) between loxP sites (IgHKO-SM), and the recombined version (IgHKO). Roosters carrying the Cre recombinase transgene were bred to hens carrying IgHKO-SM. The numbers *1*, *2*, *3* and *4* indicate the primers that were used to evaluate the recombination in (**c**). **c** Offspring of the three Cre lines were evaluated for the ability of Cre to excise floxed transgenes. *Lanes a*: birds that are positive for Cre recombinase but do not carry the IgHKO transgene. *Lanes b*: birds that are positive for Cre recombinase show the recombined version of the IgHKO transgene. *Lanes c*: birds negative for the Cre recombinase have the non-recombined IgHKO-SM transgene. **d** A schematic showing the light chain immunoglobulin knock-in locus containing a selectable marker cassette (SM) between loxP sites (IgLKI-SM), and the recombined version (IgLKI). Roosters carrying IgLKI-SM were bred to hens carrying the Cre recombinase transgene. The numbers *2*, *5*, *6* and *7* indicate the primers that were used to evaluate the recombination in (**e**). **e** Offspring from Cre hens crossed to IgLKI-SM carrying roosters. Hens from three Cre lines were evaluated for their ability to excise floxed transgenes. *Lanes a*: birds that are positive for Cre recombinase but do not carry the IgLKI transgene. *Lanes b*: birds that are positive for Cre recombinase show the recombined version of the IgLKI transgene. *Lanes c*: birds negative for the Cre recombinase also have the recombined IgLKI transgene. ctr: positive controls for the various genes/transgenes

The PGC lines selected for transfection with the Cre transgene had a stably integrated β-actin-EGFP transgene that was previously inserted using phiC31 integrase into a pseudo attP site in the genome. This EGFP transgene does not contain loxP sites, so is stable in the presence of Cre. Two male parental EGFP lines (WL527 and 169-4) were co-transfected with circular β-actin-Cre and CMV-integrase constructs (Fig. 1a). An attB site was included on the Cre plasmid to allow site-specific recombination with a pseudo-attP site in the genome, facilitated by phiC31 integrase. From each parental cell line, four stably transfected clones were expanded and confirmed for Cre integration by PCR and protein expression by immunocytochemistry (data not shown).

### Production of chimeras and transgenics

Each stably transfected clone was injected into the vasculature of 30 Stage 14–16 (H&H) embryos. A total of 123 chicks (51 % of injected embryos) hatched and were sexed by PCR for the presence of the W-chromosome. Females were euthanized and cohorts of putative male chimeras were grown to sexual maturity. Because the PGCs express the EGFP transgene, sperm of roosters within a cohort was analyzed by FACS and PCR to determine the roosters with the highest proportion of PGC-derived sperm in their semen. The selected roosters were bred and their offspring evaluated for the presence of GFP to determine the level of germline transmission. Germline transmission varied with the parental cell line used but also varied within clones from the same parental cell line (Table 1). Chimeras were bred to wild type females and transgenic males and females were hatched and grown to sexual maturity.

**Table 1:** Production of chimeras from different clonal cell populations and germline transmission

Parental cell lines	Clones injected	Age of PGCs at injection (days)	Chimeras hatched	Chimeras evaluated	Chimeras tested by breeding	Offspring evaluated	Offspring with GFP	GFP transmission (%)
WL527	1563-2	145	12	5	1	100	0	0
	1563-6	156	13	7	1	157	1	0.6
	1563-8	152	12	7	1	370	1	0.2
	1570-2	145	17	6	1	120	0	0
169-4	CreC	242	18	15	2	51	21	41, 41
	CreD	256	16	10	2	195	72	20, 48
	CreE	254	17	7	1	87	39	45
	CreF	226	18	7	4	258	4	0, 0, 2, 3

### Cre recombinase excises the floxed transgene

Three independent Cre recombinase lines were evaluated for their capability to excise floxed transgenes. Male Cre birds were crossed to females carrying a floxed immunoglobulin heavy chain knock-out transgene (Schusser et al. 2013) and progeny were analyzed for presence of Cre, the non-recombined transgene, and the recombined (looped-out) version of the transgene. Figure 1b shows the heavy chain immunoglobulin knock-out locus with a selectable marker cassette (SM) before (IgHKO-SM) and after (IgHKO) recombination of the *lox*P sites. Figure 1c shows that all offspring carrying Cre recombinase and the IgHKO transgene have the excised version of the transgene, demonstrating that excision of the selectable marker cassette from the IgHKO transgene is facilitated by the three lines CreC, CreD and CreE. In principle, primers used to detect the recombined IgH KO could also amplify the non-recombined transgene, although the product would be very large (7.1 kb).

Female Cre birds were crossed to males carrying a floxed immunoglobulin light chain knock-in transgene and progeny were analyzed for presence of Cre, the non-recombined transgene, and the recombined version of the transgene. Figure 1d shows the light chain immunoglobulin knock-in locus with a selectable marker cassette (SM) before (IgLKI-SM) and after (IgLKI) recombination of the *lox*P sites. Figure 1c shows that all offspring carrying the IgLKI transgene have the excised version of the transgene, irrespective of the presence of the Cre transgene.

### Health and reproductive performance of Cre recombinase expressing birds

**Fig. 2:**
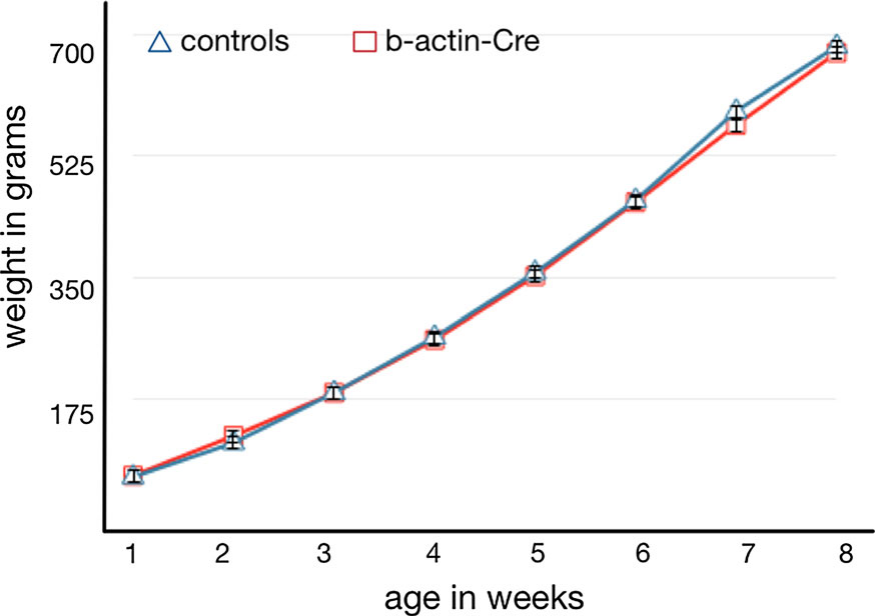
Growth between weeks 1–8 after hatch of Cre recombinase birds and age-matched controls

In a flock of 54 Cre-expressing birds that were kept for experimental purposes for up to 5 months, no morbidity nor mortality was seen. Growth of the Cre recombinase expressing birds was normal compared to age matched controls (Fig. 2). Both male and female Cre-transgenic chickens reached sexual maturity at normal times, 15 weeks for roosters and 18–20 weeks for females. CreC and CreD hemizygous males were tested for reproductive performance, and both fertility and hatching rates were normal (Table 2). Of the 94 offspring, 42 carried the Cre transgene while 52 were negative. Chi-square analysis showed that there was no deviation of the expected 50:50 ratio for inheritance of the transgene (*P* > 0.05).

**Table 2:** Reproductive performance of Cre-recombinase transgenic roosters

Males	# Eggs set	Fertility	ED	Hatched	Cre positive birds	Cre negative birds
CreC	39	37 (95 %)	0	32 (86 %)	14	18
CreD	72	67 (93 %)	3 (4 %)	62 (93 %)	28	34

*ED* embryonic death

### Chromosomal locations and linkage of Cre and GFP insertions

Since the GFP and Cre transgenes were independent integration events, we expected them to segregate independently and anticipated an equal number of GFP-expressing and non-expressing Cre offspring from matings of Cre chimeras to wild type hens. While the expected ratio was observed for CreC, all of the CreD offspring, and 7 of the 8 CreE offspring, expressed GFP (see Table 3). This linkage was confirmed for CreD in transgenic breedings of CreD hemizygous birds: all 28 offspring carrying CreD were GFP positive, while the 34 CreD negative offspring were GFP negative.

**Table 3:** Linkage of GFP and Cre transgenes in offspring obtained after breeding Cre-chimeras to wild type hens

Genotype	# Offspring	GFP positive	GFP negative
CreC	8	3	5
CreD	19	19	0
CreE	8	7	1

**Fig. 3:**
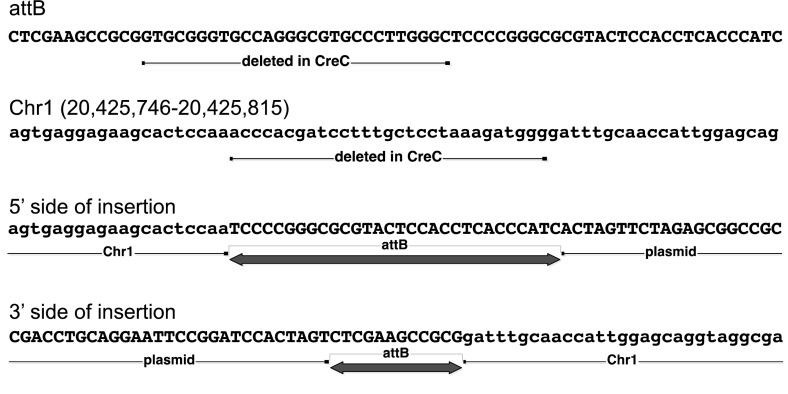
CreC genomic insertion site. The structure of the insertion site in line CreC is shown at the nucleotide level. Four sequences are shown, as follows: (1) attB: the sequence of the attB site in construct BN-B-act-Cre-attB. The full attB site is shown. A partial deletion of the attB site occurred in the CreC insertion, as indicated. (2) Chr1: sequence of the region of chicken chromosome 1 where the transgene inserted in CreC. A small deletion of the genome occurred, as indicated. (3) 5′ side of insertion: the sequence of the transgene insertion on the 5′ side (the side closest to the plasmid backbone), showing the genome/transgene junction. (4) 3′ side of insertion: the sequence of the transgene insertion on the 3′ side (the side closest to Cre), showing the genome/transgene junction

Plasmid rescue revealed that the GFP transgene in the parental line 169-4 is located in a simple tandem repeat, similar to CNM or PO41 repeats (Wicker et al. 2005) and thus the chromosomal location could not be resolved. CreC is located on Chromosome 1 in a region of non-repetitive sequences (See Fig. 3). Plasmid rescue of CreD and CreE could not be resolved but both genes must be located near the EGFP insertion in the parental line 169-4. Since the Cre and EGFP transgenes did not segregate in the offspring, no samples were available that contained only the Cre transgene. In principle both insertions should be recovered from cells that contain two independent insertions but, for unknown reasons, the Cre insertions were not amenable to plasmid rescue.

## Materials and methods

### Construct

The Cre construct consisted of a modified chicken β-actin promoter driving the Cre recombinase gene and a separate modified β-actin promoter driving expression of the neomycin resistance gene (Fig. 1a). The modified promoters consist of the chicken β-actin promoter, intron sequences from chicken β-actin and rabbit β-globin, exon sequence from rabbit β-globin, and are similar to the CAG promoter without the CMV IE enhancer. An attB site was included on the plasmid to allow site-specific recombination with a pseudo-attP site in the genome, facilitated by phiC31 integrase (Leighton et al. 2008).

### Culture and transfection of PGCs

Primordial germ cells were cultured as described previously (Van De Lavoir et al. 2012; Song et al. 2014; Collarini et al. 2015). Briefly, primordial germ cells were grown in KO-DMEM (Life Technologies), of which 40 % was preconditioned on buffalo rat liver cells (BRL, ATCC, Catalogue number CRL-12442; Van De Lavoir and Mather-Love 2006), and supplemented with 7.5 % fetal bovine serum (Hyclone), 2.5 % chicken serum, 1X non-essential amino acids, 2 mM glutamine, 1 mM sodium pyruvate, 0.1 mM β-mercaptoethanol (all from Life Technologies), 4 ng/ml recombinant human fibroblast growth factor, 6 ng/ml recombinant mouse stem cell factor (both from R&D Systems) and grown on an irradiated feeder layer of BRL cells. The cells were passaged 3 times per week onto fresh feeder layers.

#### Transfection of primordial germ cells

Integrase-mediated transfection was used to obtain stably expressing β-actin-Cre cell lines (Leighton et al. 2008). Integrase facilitates site-specific recombination between the attB site on the β-actin-Cre construct and a pseudo-attP site in the genome (Groth et al. 2000; Thyagarajan et al. 2001). Two male parental cell lines containing a stably-integrated, ubiquitously-expressed β-actin-EGFP transgene were chosen as starting cells to allow tracking of the cells to the germline and in the sperm.

For each transfection, 7.5 µg of β-actin-Cre plasmid and 15 µg of CMV-integrase plasmid (Thyagarajan et al. 2001) were added to 5 × 10^6^ cells and brought to a volume of 100 ul with V-buffer (Lonza, Walkersville). The cell suspension was transferred to a 2 mm cuvette and subjected to 8 square wave pulses of 350 V/100 µs (BTX 830 electroporator). After electroporation the cells were resuspended in culture medium with neomycin-resistant irradiated BRLs and seeded in a 48-well plate at a density of 10^5^ cells per well. After 4 days, 350 µg/ml geneticin (Teknova) was added to select for cells with a stable integration of the β-actin-Cre/β-actin-Neo construct. After stable clones were identified, the cells were expanded and confirmed for Cre integration by PCR and protein expression by immunocytochemistry using a monoclonal antibody against Cre protein (Millipore). Confirmed clones were injected into recipient chicken embryos at Stage 14–16 (H&H). The injected embryos were transferred to surrogate shells and incubated until hatch at 37 °C. The sex of the chicks was determined after hatch by PCR for the W-chromosome.

#### Localization of the integration

The location of the Cre and GFP transgenes in the genome was determined by plasmid rescue. Genomic DNA was purified from transgenic animals or PGCs and digested with an enzyme that does not cut in the transgene. Fragments were ligated under dilute conditions to favor re-circularization of linear pieces, and transformed into DH10B electrocompetent cells. Ampicillin-resistant colonies were obtained, derived from plasmid DNA integrated into the chicken genome. Sequencing of the junction between the vector and the flanking genomic sequence confirmed that the insertion occurred via the attB site on the vector and was thus integrase-mediated. The flanking genomic sequence was subjected to BLAST and BLAT to map the insertions to the genome database.

#### Evaluation of chimeras

Male chimeras were grown to sexual maturity and their semen was analyzed by FACS and by PCR. For FACS analysis the sperm was diluted with PBS/0.5 % BSA and filtered through strainer caps. The chimeras with the highest percentage of GFP-positive sperm were then bred to wild type females. The eggs were incubated and evaluated at E7 for the presence of GFP-positive embryos. Progeny from the best germline transmitters were then hatched and grown to sexual maturity.

### Evaluation of hemizygous Cre recombinase expressing birds

#### Growth characteristics

Weights from 20 β-actin-Cre birds and 20 age-matched controls were taken once a week for 8 weeks. Morbidity and mortality of birds carrying the Cre transgene were recorded and evaluated.

#### Evaluation of Cre activity and recombination of the transgenes

Functionality of the Cre gene was evaluated in offspring from matings between roosters carrying β-actin-Cre and hens containing an immunoglobulin heavy chain knock out with a floxed selectable marker cassette (Schusser et al. 2013; Fig. 1b) and between hens carrying β-actin-Cre and roosters containing an immunoglobulin light chain knock-in with a floxed selectable marker cassette (Fig. 1d). Progeny of these crosses were genotyped for Cre and the floxed transgene. The GFP expression was determined by visual examination using a head set containing a UV light source and goggles that visualize EGFP (BLS, Budapest, Hungary). Combs were removed from the chicks at hatch and PCR specific for the non-recombined (IgHKO-SM or IgLKI-SM) and the recombined (IgHKO or IgLKI) versions of the transgene was performed using primers as indicated in Fig. 1b, d. Primer 1: chDJ-F7 5′-TGAACCCATAAAGTGAAATCCTC-3′. Primer 2: HA-R 5′-ATACGATGTTCCAGATTACGCTT-3′, product of Primers 1 + 2 = 572 bp; Primer 3: chDJ-F10 5′-AATTTGGAGCGAAGGATGGC-3′. Primer 4: neo-F2 5′-GGTTCGAAATGACCGACCAAGC-3′, product of Primers 3 + 4 = 589 bp. Primer 5: L-40303-F 5′-ACTGTGCTGCAGGTGGCTATG-3′, product of primers 5 + 2 = 1145 bp. Primer 6: L-41282-F 5′-AGATCTCCTCCTCCCATCCTG-3′; Primer 7: S14B 5′-TCCTCTCTCTTCTTAACCAC-3′; product of Primers 6 + 7 = 587 bp. Primers for Cre were: CreSeq-F2 5′-TATGCGGCGGATCCGAAAAG-3′: and Cre-R2 5′-CTTCCAGGGCGCGAGTTGAT-3′: product size = 440 bp.

## Discussion

Three lines of birds (CreC, CreD and CreE) were produced from PGCs carrying Cre recombinase driven by the β-actin promoter. In transgenic animals, expression levels were sufficient to catalyze excision of DNA sequences flanked by loxP sites.

The parental source of the Cre recombinase affected the recombination of the floxed transgenes. When the Cre recombinase was expressed in the father, only offspring that inherited the Cre recombinase gene showed recombination of the floxed transgene. However, when the Cre recombinase was expressed by the mother, recombination of the floxed transgene occurred in all offspring. The latter indicates that sufficient amounts of the Cre recombinase protein are present in the oocyte of the egg to recombine the floxed transgenes present in the sperm genome. Similar activity of Cre recombinase in oocytes has been reported in mice (Lallemand et al. 1998). The β-actin promoter that drives Cre recombinase expression is active in primordial germ cells. We do not know if it is active when the germ cells differentiate but the activity of Cre recombinase in the cytoplasm suggests the promotor is active in the germinal vesicle. Initiated by the preovulatory surge of luteinizing hormone, the first meiotic division results in expulsion of the first polar body within 1 h of ovulation, at which time the transgene either remains in the oocyte or is expelled (Etches 1996). Shortly after ovulation, spermatozoa penetrate the oocyte and the male pronuclei are released into the egg. One of the male pronuclei will fuse with the female pronucleus within 4 h after fertilization at which time mitotic divisions start. There is a period of approximately 4–5 h between the expulsion of the Cre recombinase transgene into a polar body and the fusion of the male and female pronuclei. The results indicate that there is enough Cre recombinase present in the cytoplasm surrounding the nucleus at that time to recombine the transgenes present in the male genome.

The presence of Cre recombinase in the oocyte is advantageous because all of the F1 offspring carry the recombined transgene and half of them do not carry the Cre transgene. In contrast when Cre recombinase is introduced using a rooster carrying Cre, the recombined transgenes are only present in offspring that carry the Cre transgene. To eliminate the Cre transgene, an F2 generation is required.

Off-target effects of the human and mouse genomes by Cre recombinase have been observed in vitro and in vivo (Thyagarajan et al. 2000; Schmidt et al. 2000; Loonstra et al. 2001) and can lead to health and reproductive abnormalities. In the CreC, CreD and CreE birds normal growth rates and viability indicate that there are no obvious adverse effect of Cre expression in these birds. Both male and female Cre-expressing chickens reached sexual maturity at the expected age and showed normal reproductive behavior as indicated by high levels of fertility and hatching of offspring derived from both the males and the females.

Unexpectedly, the integration of the Cre recombinase transgene in the CreD and CreE lines was genetically very tightly linked to the GFP transgene integration, indicating that there is a ‘hotspot’ in the chicken genome where site-specific recombination facilitated by integrase is preferred. As a result we have created three different Cre recombinase expressing lines; CreD and CreE that are linked to GFP expression, and CreC that is not. Plasmid rescue experiments were not able to identify the chromosome where the GFP integrated, but did indicate that the integration happened in a repeat sequence similar to PO41. The GFP expressing line allows for very easy selection at hatch for chicks carrying Cre recombinase while the non-GFP line would be useful in experiments where GFP expression interferes with analysis.

These data demonstrate for the first time that ubiquitous expression of Cre recombinase can be used in birds to remove floxed sequences. Combining Cre coding sequences with tissue-specific promoters opens a myriad of opportunities for chickens with developmentally-regulated and tissue-specific expression of transgenes in the future. The presence of Cre recombinase in the oocyte’s cytoplasm allows timely evaluation of the transgene without any potential negative effects from selectable markers.
